# Mutual causal effects between immune cells and hepatocellular carcinoma: a Mendelian randomization study

**DOI:** 10.1007/s12672-025-01785-z

**Published:** 2025-01-16

**Authors:** Zheng Wang, Mengshu Pan, Jie Zhu, Changhong Liu

**Affiliations:** 1 Department of Critical Care Medicine, Shandong Provincial Hospital Affiliated to Shandong First Medical University, Jinan, 250021 Shandong China; 2 Primary Care Department, Second Affiliated Hospital of Anhui Medical University, Hefei, 230000 Anhui China; 3 Department of Infectious Disease, Second Affiliated Hospital of Anhui Medical University, Hefei, 230000 Anhui China; 4 Department of Gastroenterology, The First Affiliated Hospital of Shandong First Medical University & Shandong Provincial Qianfoshan Hospital, Jinan, 250014 Shandong China

**Keywords:** Immune cell, Hepatocellular carcinoma, Mendelian randomization, Immunophenotypes

## Abstract

**Background:**

Hepatocellular carcinoma (HCC), a malignant tumor that seriously endangering health, has aroused widespread concern in the field of public health. Previous researches have noted the relationships between immune cells and HCC, but the causal relationship was uncertain.

**Methods:**

In this study, a bidirectional two sample Mendelian randomization (MR) analysis was utilized to access the causal relationship between immune cell characteristics and HCC. According to the open-access data, we investigated the causal relationship between 731 immune cell characteristics and HCC risk.

**Results:**

After screening by IVW approach, increased levels of 8 immune traits and reduced levels of 7 immune traits could lead to changes in HCC risk. These 15 immune cells were distributed in the Monocyte (4 cells), Treg panel (4 cells), TBNK (3 cells), Maturation stages of T cell panel (3 cells), and cDC panel (1 cells). Furthermore, HCC was identified to have causal effects on 21 immunophenotypes. Among these immune cells, hepatocarcinogenesis had the greatest impact on CD4 on EM CD4 + and CD33 on Mo MDSC.

**Conclusions:**

This study enhances our comprehension of the interaction between immune cells and HCC risk, furnishing novel avenues to explore the mechanisms of HCC.

**Supplementary Information:**

The online version contains supplementary material available at 10.1007/s12672-025-01785-z.

## Introduction

Hepatocellular carcinoma (HCC) is an aggressive malignancy that remains one of the most common causes of cancer-relevant death worldwide [1]. Although the therapeutic measures, including surgical resection, have been improved, the 5-year survival rate for HCC patients is still disappointing [2]. HCC is a multifactorial illness with numerous underlying causes [3]. Genetic and immune environmental factors participate in its occurrence and progress, yet the specific mechanisms remain unclear.

The liver, as a central immunological organ, has a large number of immune cells that are exposed to various microbial antigens via the gut-liver axis [4]. Moreover, the immune cell composition of HCC tumor microenvironment possesses a significant influence on tumor biology [5]. Many studies have confirmed a bidirectional relationship between HCC and immune cells. NK cells are the main components of innate immune cells in the liver, playing a core role in innate immunity [6]. NK cells play a double-edged role in chronic HBV infection [7]. NK cells possess anti-fibrosis function [8], while NK cell activation mediates HBV-related liver cell injury, thereby promoting HCC development [9]. Dendritic cells (DCs) are specialized antigen-presenting cells that play a prominent role in starting immune responses [10]. DCs play a complex role in the occurrence and development of HCC [11]. On the one hand, DCs could fight against HCC by activating T cell responses [12]. On the other hand, regulatory DCs could inhibit anti-tumor immunity and promote tumor immune escape through indoleamine-2,3-dioxygenase [13]. Nevertheless, most of the existing evidence was according to observational studies, which may have restrictions owing to confounding variables and reverse causation.

The causal associations between immune cell characteristics and HCC have not been well elucidated.

Mendelian randomization (MR) is an emerging analytical approach utilized for causal inference of risk factors and disorders in epidemiology [14]. Single nucleotide polymorphisms (SNPs) were utilized as an instrumental variable to unveil the causality of the correlation between exposure and outcome. SNPs follow the criterion of random genetic variation distribution during meiosis, eliminating the effect of mixed factors and the underlying effect of reverse causality [15]. Former researches have illustrated that there were plenty of correlations between immune cell characteristics and various diseases, confirming the hypothesis of associations among them [16, 17]. In this research, a comprehensive two-sample MR approach was conducted to investigate the causal relationship between 731 immune cell characteristics and HCC.

## Materials and methods

### Study design

According to the massive amounts of GWAS data, we evaluated the bi-directional causal relationships between a great quantity of immune traits (731 immunophenotypes in seven groups [18]) and hepatocellular carcinoma risk via a two-sample Mendelian randomization method. To guarantee effective instrumental variable estimation in MR analyses, three principal assumptions must be satisfied [19]. First, the selected SNPs should be intimately and strongly connected to exposure factors. Second, the SNPs ought to be independent of any potential confounding elements. Last but not least, the SNPs only influences the outcome via exposure variables and are not affected by other ways.

### Data sources

We selected the SNPs related to hepatocellular carcinoma (HCC) from the IEU Open GWAS project (https://gwas.mrcieu.ac.uk/datasets/bbj-a-158/), which conducted a genome-wide association study (GWAS) on 197,611 East Asian individuals (Ncase = 1866, Ncontrol = 195,745). The dataset included approximately 8,885,115 SNPs. This study was chosen due to its large sample size, specifically for East Asian populations, and its focus on hepatocellular carcinoma, making it highly relevant for our analysis of genetic risk factors for HCC. The selection of this dataset ensures that our analysis is based on a well-powered GWAS with a large control group, minimizing the risk of confounding bias [20].

Additionally, we extracted the GWAS summary statistics for 731 immune cell phenotypes from the GWAS Catalog (accession numbers from GCST90001391 to GCST90002121). These 731 immunophenotypes were classified into four broad categories: 389 median fluorescence intensities (MFI), 192 relative cell counts (RC), 118 absolute cell counts (AC), and 32 morphological parameters (MP). We chose this dataset because it provides detailed immune cell measurements that are crucial for understanding the immune landscape in relation to HCC risk. These phenotypes are comprehensive, encompassing both quantitative and morphological immune traits, which are essential for capturing the immune profile associated with hepatocellular carcinoma.

For the association analysis, approximately 22 million high-quality markers were retained after quality control. We performed the analysis by adjusting for gender, age, and age2 as covariates, to account for potential confounding effects and improve the precision of our findings [21].

### Instrument selection

Given a quantity of single nucleotide polymorphisms (SNPs) reaching genome-wide significance (p < 1 × 10^−5^) for 731 immunophenotypes, we adopted a more stringent correlation threshold (p < 5 × 10^−8^) as the inclusion criteria. SNPs (r2 > 0.001 within 10,000 kilobases) with palindromic structure were excluded. SNPs with F statistics fewer than 10 were identified as weak tools and also excluded (Table 1). In order to assess causal link between 731 immune traits and HCC, inverse variance weighting (IVW) [22], MR Egger and Weighted median were chiefly implemented via the ‘TwoSampleMR’ package (version 0.5.7). The Q statistic of Cochran and the homologous p values were utilized to measure the heterogeneity between the chosen IVs. In order to avoid the instrumental variables affecting the outcome through other factors than the exposure factors, we analyzed the pleiotropy of the instrumental variables via MR-Egger intercept. Only the absence of pleiotropy could indicate the robustness of the results. The results were represented by estimated values (odds ratio (OR) and its 95% confidence interval (CI)), and plotted using a forest map. All analyses were implemented via the TwoSampleMR package in R (version 4.3.1). A two-sided p < 0.05 was considered statistically significant.

**Table 1:** Instrumental variables used in MR analysis of the association between immune cell traits and HCC risk

Exposure		SNP	Chr	Position	Exposure			Outcome			F-value
Cell type	Characteristics				Beta	SE	P-value	Beta	SE	P-value	
cDC	CD11c on granulocyte	rs113861918	19	52129097	− 0.215	0.037	6.42E-09	− 0.059	0.047	0.208	33.888
		rs1801274	1	161479745	0.248	0.030	1.36E-16	0.098	0.041	0.017	431.123
		rs28432694	16	48666310	− 0.172	0.030	1.75E-08	− 0.006	0.038	0.879	31.910
Maturation stages of T cell	CD8 on EM CD8br	rs17201560	6	32047268	− 0.519	0.086	2.13E-09	− 0.081	0.108	0.450	36.053
		rs2571390	6	29923554	0.364	0.038	6.16E-22	0.089	0.054	0.096	94.109
		rs3020726	2	87016506	− 0.298	0.040	7.15E-14	− 0.067	0.075	0.373	194.449
	Naive CD4 + %CD4 +	rs113243185	6	32585071	− 0.210	0.036	8.06E-09	0.039	0.045	0.391	62.639
		rs12712610	2	38897249	0.146	0.025	1.18E-08	− 0.069	0.034	0.043	68.111
		rs9271536	6	32589978	0.232	0.033	3.60E-12	− 0.115	0.039	0.004	76.551
	TD DN (CD4-CD8-) %T cell	rs111983490	6	32205324	− 0.217	0.037	3.74E-09	0.008	0.044	0.846	62.119
		rs6729180	2	38920621	0.202	0.026	1.78E-14	− 0.075	0.033	0.026	60.235
		rs6755452	2	87035076	0.190	0.032	4.75E-09	− 0.050	0.075	0.506	51.878
Monocyte	CD40 on CD14 + CD16- monocyte	rs1801274	1	161479745	0.437	0.026	1.62E-62	0.099	0.041	0.017	431.123
		rs60629714	9	95889160	0.186	0.032	4.55E-09	0.0006	0.040	0.988	34.513
		rs745307	20	44747086	0.664	0.033	2.07E-83	0.024	0.088	0.787	458.015
	CX3CR1 on CD14 + CD16- monocyte	rs1801274	1	161479745	− 0.450	0.026	2.10E-64	0.098	0.041	0.017	431.123
		rs72865957	3	39355919	0.283	0.029	6.00E-23	− 0.110	0.069	0.111	98.618
		rs9823718	3	39293757	− 0.412	0.037	5.78E-29	0.104	0.059	0.081	126.907
	CX3CR1 on CD14 + CD16 + monocyte	rs1801274	1	161479745	− 0.463	0.026	2.62E-69	0.098	0.041	0.017	431.122
		rs2649752	3	39351805	0.158	0.029	3.51E-08	− 0.111	0.069	0.108	30.499
		rs9823718	3	39293757	− 0.327	0.037	5.15E-19	0.103	0.059	0.081	126.907
	CX3CR1 on monocyte	rs1801274	1	161479745	− 0.410	0.026	4.45E-54	0.099	0.041	0.017	431.123
		rs72865957	3	39355919	0.239	0.028	6.28E-17	− 0.110	0.069	0.111	98.617
		rs9823718	3	39293757	− 0.408	0.036	7.85E-29	0.104	0.059	0.081	126.907
TBNK	FSC-A on CD4 +	rs13191523	6	31009094	0.277	0.046	1.73E-09	− 0.162	0.309	0.599	36.449
		rs7745305	6	30913475	0.206	0.038	4.51E-08	0.072	0.218	0.739	30.063
		rs9271673	6	32592833	− 0.209	0.033	1.93E-10	− 0.117	0.039	0.003	40.784
	SSC-A on CD4 +	rs113243185	6	32585071	0.240	0.037	1.39E-10	0.038	0.045	0.391	62.639
		rs148031710	6	31148873	0.297	0.049	1.91E-09	− 0.145	0.548	0.791	36.264
		rs79493037	6	31405825	0.229	0.041	2.31E-08	0.081	0.066	0.220	31.354
		rs9271536	6	32589978	− 0.259	0.035	8.85E-14	− 0.114	0.039	0.004	76.551
	SSC-A on granulocyte	rs2236947	3	50371432	0.181	0.028	2.24E-10	0.066	0.039	0.097	40.494
		rs2738144	8	6826043	− 0.246	0.029	1.59E-17	− 0.016	0.057	0.772	73.405
		rs42031	7	92237396	− 0.239	0.030	5.55E-15	− 0.067	0.081	0.407	61.636
		rs445	7	92408370	0.437	0.032	3.84E-40	0.057	0.035	0.120	180.877
Treg	CD28- CD8br %CD8br	rs4796089	17	33814758	− 0.203	0.033	1.35E-09	0.085	0.038	0.027	43.052
		rs6904670	6	31192797	− 0.146	0.026	3.34E-08	0.059	0.035	0.088	30.622
		rs7728865	5	59819941	− 0.217	0.038	9.95E-09	− 1.051	1.667	0.528	33.011
	CD39 on granulocyte	rs11766311	7	77134132	− 0.153	0.028	4.55E-08	− 0.072	0.035	0.042	30.046
		rs445	7	92408370	0.209	0.034	1.06E-09	0.057	0.035	0.110	180.877
		rs7073937	10	97469109	− 0.204	0.034	2.45E-09	− 0.046	0.052	0.375	35.786
	CD127 on CD45RA + CD4 +	rs11567754	5	35872190	− 0.298	0.043	8.08E-12	0.029	0.052	0.583	47.087
		rs1573735	16	423696	− 0.207	0.028	4.02E-13	− 0.017	0.036	0.638	53.075
		rs3129891	6	32415080	− 0.237	0.034	5.88E-12	0.102	0.035	0.004	47.719
		rs9271772	6	32594275	0.261	0.035	2.23E-13	− 0.114	0.039	0.004	54.239
	CD8 on CD39 + CD8br	rs111359007	6	31773975	− 0.280	0.048	5.34E-09	0.960	1.415	0.498	34.238
		rs117519014	10	97403248	0.435	0.066	7.57E-11	2.090	1.015	0.040	42.658
		rs17197199	6	31138472	− 0.284	0.043	7.21E-11	− 0.031	0.042	0.455	42.742
		rs72847706	2	87055981	− 0.255	0.032	1.80E-15	− 0.075	0.054	0.168	63.946
		rs9357097	6	30285121	0.192	0.028	8.08E-12	0.065	0.033	0.046	47.099

*SNP* single nucleotide polymorphism, *Chr* Chromosome, *SE* Standard Error

## Results

### Investigation of the causal impact of immune cells on HCC risk

**Fig. 1:**
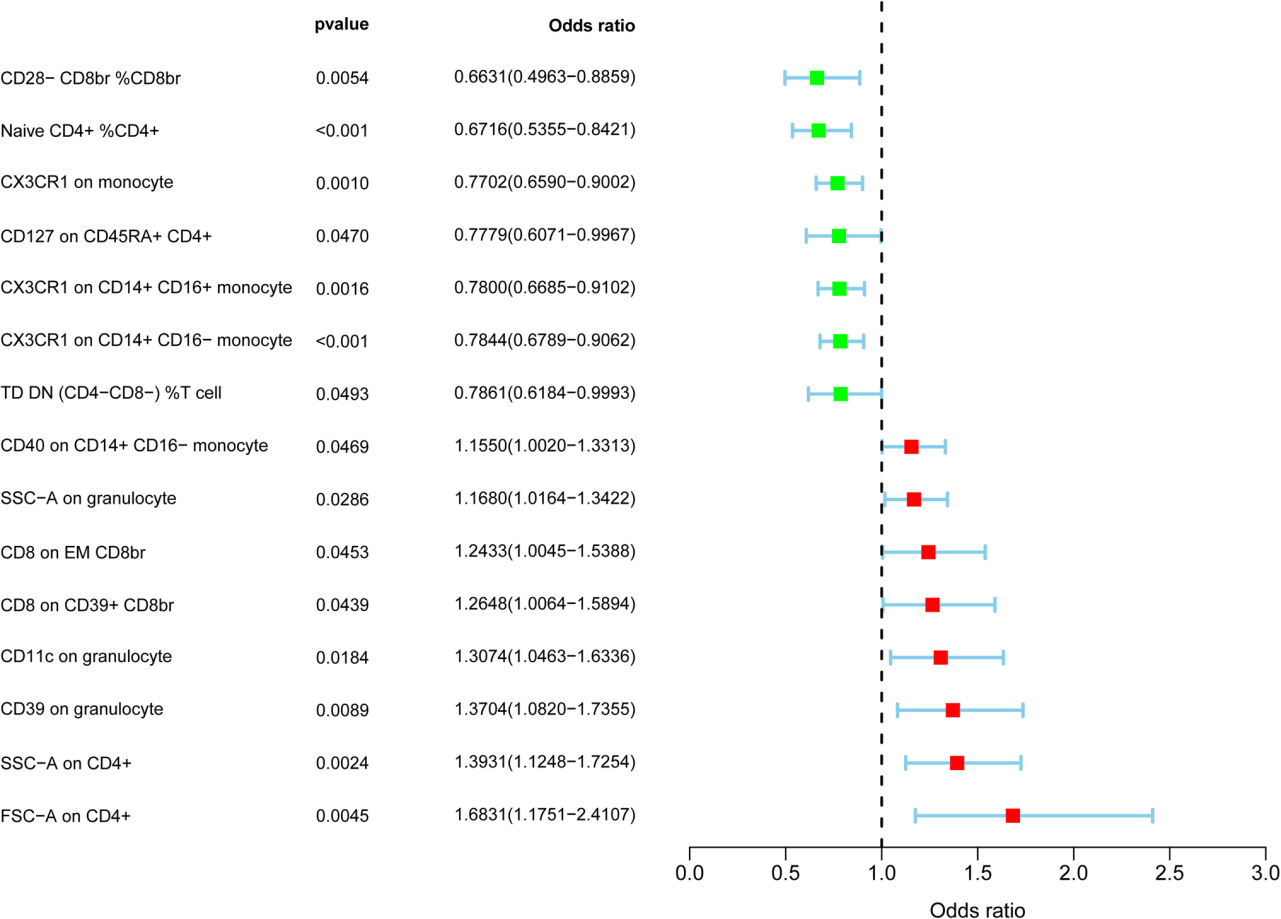
Forest plots showed the causal associations between immune cell traits and HCC by using IVW method

In order to investigate the causal impact of immune cells on HCC risk, two-sample MR Analyses were carried out, and the IVW approach was occupied as the primary analysis. After screening by IVW approach, increased levels of 8 immune traits and reduced levels of 7 immune traits could lead to changes in HCC risk (Fig. 1). 15 immune cells were detected, including 4 in the Monocyte and Treg panel, 3 in the TBNK and Maturation stages of T cell panel, and 1 in the cDC panel (Table 2).

**Table 2:** MR results for causal impact of immune cells on HCC risk

Exposure		Method	No SNP	OR (95%CI)	*P*-Value
Cell type	Characteristics				
cDC	CD11c on granulocyte	IVW	3	1.31 (1.05 to 1.63)	**0.018**
		Weighted median		1.36 (1.03 to 1.79)	**0.029**
		Weighted mode		1.42 (1.00 to 2.02)	0.192
		Simple mode		1.38 (1.94 to 2.04)	0.244
		MR Egger		3.39 (0.81 to 14.18)	0.343
Maturation stages of T cell	CD8 on EM CD8br	IVW	3	1.24 (1.00 to 1.54)	**0.045**
		Weighted median		1.26 (1.00 to 1.59)	0.053
		Weighted mode		1.27 (0.96 to 1.69)	0.234
		Simple mode		1.27 (0.94 to 1.70)	0.260
		MR Egger		1.06 (0.33 to 3.41)	0.935
	Naive CD4 + %CD4 +	IVW	3	0.67 (0.54 to 0.84)	**0.001**
		Weighted median		0.62 (0.47 to 0.83)	**0.001**
		Weighted mode		0.61 (0.43 to 0.88)	0.119
		Simple mode		0.62 (0.42 to 0.90)	0.132
		MR Egger		0.73 (0.19 to 2.75)	0.722
	TD DN (CD4-CD8-) %T cell	IVW	3	0.79 (0.62 to 0.99)	**0.049**
		Weighted median		0.75 (0.56 to 1.00)	**0.046**
		Weighted mode		0.70 (0.49 to 0.99)	0.188
		Simple mode		0.74 (0.49 to 1.12)	0.288
		MR Egger		15.53 (0.05 to 5008.70)	0.522
Monocyte	CD40 on CD14 + CD16- monocyte	IVW	3	1.15 (1.00 to 1.33)	**0.047**
		Weighted median		1.15 (0.98 to 1.33)	0.078
		Weighted mode		1.25 (1.05 to 1.50)	0.135
		Simple mode		1.02 (0.79 to 1.32)	0.894
		MR Egger		1.21 (0.77 to 1.89)	0.559
	CX3CR1 on CD14 + CD16- monocyte	IVW	3	0.78 (0.68 to 0.91)	** < 0.001**
		Weighted median		0.79 (0.68 to 0.93)	**0.003**
		Weighted mode		0.80 (0.68 to 0.94)	0.115
		Simple mode		0.79 (0.64 to 0.97)	0.158
		MR Egger		1.07 (0.42 to 2.76)	0.909
	CX3CR1 on CD14 + CD16 + monocyte	IVW	3	0.78 (0.67 to 0.91)	**0.002**
		Weighted median		0.79 (0.67 to 0.93)	**0.004**
		Weighted mode		0.80 (0.67 to 0.94)	0.115
		Simple mode		0.77 (0.61 to 0.97)	0.161
		MR Egger		1.04 (0.63 to 1.73)	0.899
	CX3CR1 on monocyte	IVW	3	0.77 (0.66 to 0.90)	**0.001**
		Weighted median		0.78 (0.66 to 0.93)	**0.004**
		Weighted mode		0.78 (0.65 to 0.95)	0.126
		Simple mode		0.78 (0.63 to 0.96)	0.148
		MR Egger		1.06 (0.44 to 2.58)	0.917
TBNK	FSC-A on CD4 +	IVW	3	1.68 (1.18 to 2.41)	**0.005**
		Weighted median		1.72 (1.12 to 2.65)	**0.014**
		Weighted mode		1.74 (1.15 to 2.63)	0.121
		Simple mode		1.58 (0.65 to 3.82)	0.419
		MR Egger		0.017 (2.05E-06 to 147.78)	0.541
	SSC-A on CD4 +	IVW	4	1.39 (1.12 to 1.73)	**0.002**
		Weighted median		1.45 (1.12 to 1.87)	**0.004**
		Weighted mode		1.46 (1.12 to 1.90)	0.068
		Simple mode		1.42 (1.04 to 1.93)	0.112
		MR Egger		6.12 (0.077 to 487.12)	0.502
	SSC-A on granulocyte	IVW	4	1.17 (1.02 to 1.34)	**0.029**
		Weighted median		1.15 (0.99 to 1.34)	**0.073**
		Weighted mode		1.14 (0.96 to 1.34)	0.236
		Simple mode		1.14 (0.90 to 1.44)	0.353
		MR Egger		0.99 (0.67 to 1.47)	0.984
Treg	CD28- CD8br %CD8br	IVW	3	0.66 (0.50 to 0.89)	**0.005**
		Weighted median		0.66 (0.48 to 0.91)	**0.011**
		Weighted mode		0.66 (0.49 to 0.91)	0.124
		Simple mode		0.66 (0.48 to 0.91)	0.129
		MR Egger		0.65 (0.11 to 3.78)	0.716
	CD39 on granulocyte	IVW	3	1.37 (1.08 to 1.74)	**0.009**
		Weighted median		1.32 (0.99 to 1.76)	0.059
		Weighted mode		1.30 (0.94 to 1.79)	0.258
		Simple mode		1.28 (0.90 to 1.82)	0.298
		MR Egger		0.72 (0.14 to 3.69)	0.763
	CD127 on CD45RA + CD4 +	IVW	4	0.78 (0.61 to 0.99)	**0.047**
		Weighted median		0.74 (0.61 to 0.91)	**0.004**
		Weighted mode		0.67 (0.48 to 0.93)	0.097
		Simple mode		0.68 (0.45 to 1.00)	0.147
		MR Egger		0.43 (0.04 to 4.48)	0.556
	CD8 on CD39 + CD8br	IVW	5	1.26 (1.01 to 1.59)	**0.044**
		Weighted median		1.29 (1.02 to 1.64)	**0.047**
		Weighted mode		1.29 (1.04 to 1.61)	0.079
		Simple mode		1.25 (1.03 to 1.53)	0.091
		MR Egger		0.84 (0.20 to 3.46)	0.825

All statistical tests were two-sided

*MR* Mendelian randomization, *IVW* inverse variance weighted, *SNP* single nucleotide polymorphism, *OR* odds ratio, *CI* confidence intervals.

Bold values indicates the *p*-value is less than 0.05

On the Monocyte panel, odds ratio (OR) of CX3CR1 on monocyte on HCC risk was estimated to be 0.770 (95% CI 0.6589–0.900, P = 1.031 × 10^–3^) by using IVW approach. Similar result was observed by using weighted median approach (P = 3.685 × 10^–3^). OR of CX3CR1 on CD14 + CD16 + monocyte on HCC risk was estimated to be 0.780 (95% CI 0.669–0.910, P = 1.602 × 10^–3^) by using IVW approach.Similar result was observed by using weighted median approach (P = 1.602 × 10^–3^). OR of CX3CR1 on CD14 + CD16- monocyte on HCC risk was estimated to be 0.784(95% CI 0.679–0.906, P = 0.981 × 10^–3^) by using IVW approach. Similar result was observed by using weighted median approach (P = 3.589 × 10^–3^). We detected a causal efect of CD40 on CD14 + CD16- monocyte on HCC risk by using the IVW approach (OR = 1.155, 95% CI 1.002–1.331, P = 0.047). On the Treg panel, odds ratio (OR) of CD28- CD8br %CD8br on HCC risk was estimated to be 0.663 (95% CI 0.496–0.886, P = 5.434 × 10^–3^) by using IVW approach. Similar result was observed by using weighted median approach (P = 0.012). OR of CD39 on granulocyte on HCC risk was estimated to be 1.370 (95% CI 1.082–1.736, P = 8.947 × 10^–3^) by using IVW approach. OR of CD8 on CD39 + CD8br on HCC risk was estimated to be 1.265 (95% CI 1.006–1.590, P = 0.043) by using IVW approach. Similar result was observed by using weighted median approach (P = 0.022). We detected a causal effect of CD127 on CD45RA + CD4 + on HCC risk by using the IVW approach (OR = 0.778, 95% CI 0.607–0.997, P = 0.047). On the TBNK panel, OR of SSC-A on CD4 + on HCC risk was estimated to be 1.393 (95% CI 1.125–1.725, P = 2.387 × 10–3) by using IVW approach. Similar result was observed by using weighted median approach (P = 4.659 × 10–3). OR of FSC-A on CD4 + on HCC risk was estimated to be 1.683 (95% CI 1.175–2.411, P = 4.508 × 10–3) by using IVW approach. Similar result was observed by using weighted median approach (P = 0.012). OR of SSC-A on granulocyte on HCC risk was estimated to be 1.168 (95% CI 1.016–1.342, P = 0.029) by using IVW approach. On the Maturation stages of T cell panel, OR of Naive CD4 + %CD4 + on HCC risk was estimated to be 0.672 (95% CI 0.536–0.842, P = 5.654 × 10–4) by using IVW approach. Similar result was observed by using weighted median approach (P = 1.017 × 10–3). OR of CD8 on EM CD8br on HCC risk was estimated to be 1.243 (95% CI 1.005–1.539, P = 0.045) by using IVW approach. OR of TD DN (CD4-CD8-) %T cell on HCC risk was estimated to be 0.786 (95% CI 0.618–0.999, P = 0.049) by using IVW approach. On the cDC panel, OR of CD11c on granulocyte on HCC risk was estimated to be 1.307 (95% CI 1.046–1.634, P = 0.018) by using IVW approach. Similar result was observed by using weighted median approach (P = 0.022).

### Reverse validation and investigation of the causal impact of HCC onset on immunophenotypes

**Fig. 2:**
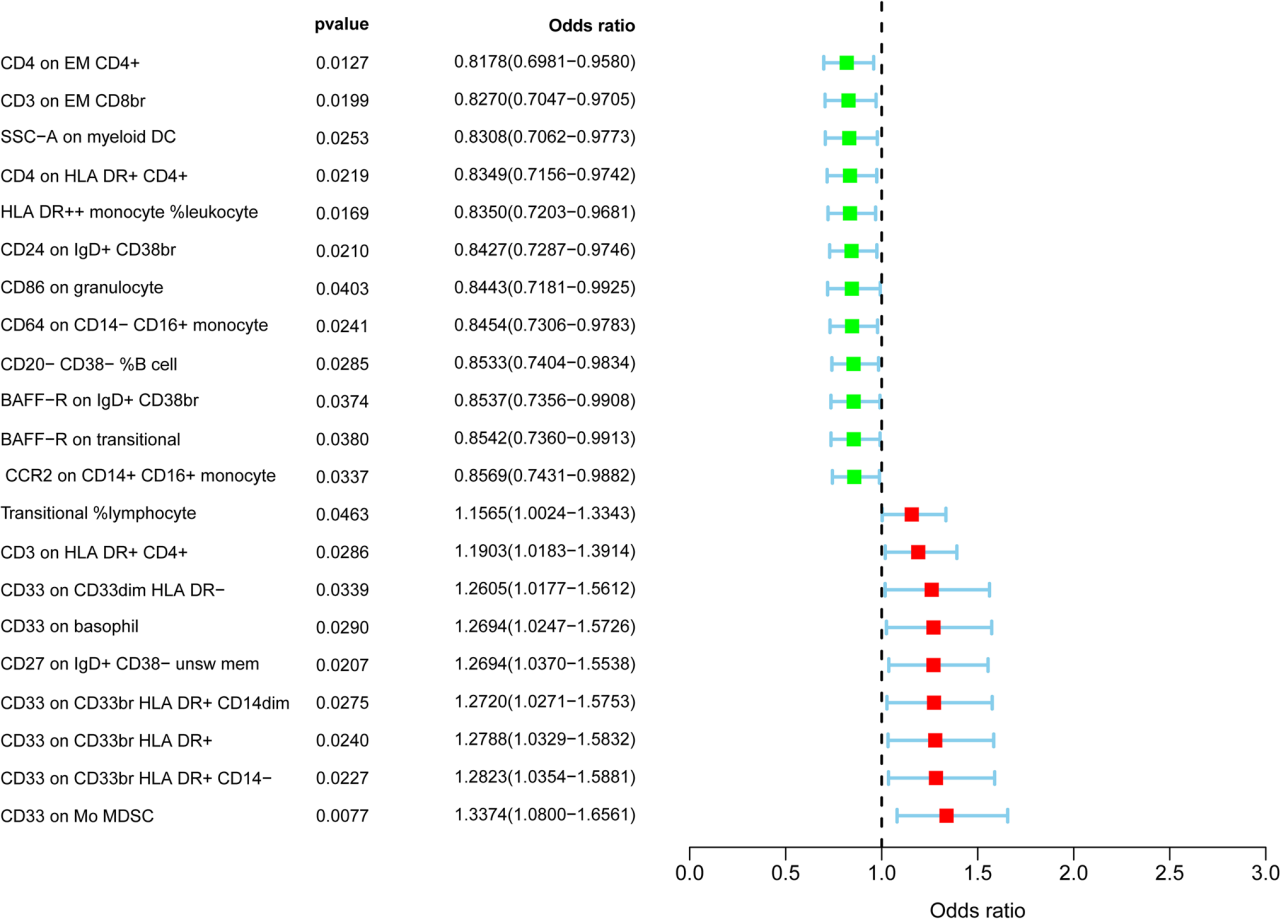
Forest plots showed the causal associations between HCC and immune cell traits

To validate the causal effect of immune cells as an exposure on the outcome—HCC risk—and further explore the impact of hepatocellular carcinoma on immune cells, we employed reverse MR. The selection of instrumental variables was performed using the same parameters as those in the forward MR (Table S1). We identified causal impact of HCC on the levels of 21 immunophenotypes, where HCC onset can increase levels of 9 immune traits and reduce levels of 12 immune traits (Fig. 2). These 21 immune cells were distributed in B cell (6 cells), cDC (2 cells), Maturation stages of T cell 2 cells), Monocyte (2 cells), Myeloid cell (6 cells), TBNK (3cells). Among these immune cells,  hepatocarcinogenesis had the greatest impact on CD4 on EM CD4 + (OR = 0.818,95% CI 0.698–0.958, P = 0.013) and CD33 on Mo MDSC (OR = 1.337,95% CI 1.080–1.656, P = 7.677 × 10 − 3), with CD4 on EM CD4 + decreasing and CD33 on Mo MDSC increasing (Table S2).

## Discussion

Our research incorporated massive amounts of GWAS data, systematically unveiling the mechanisms of immune cells in the initiation and progression of HCC from a genetic point of view. As far as we know, this is among the limited number of studies that have examined the causal association between various immune cells and HCC via MR analysis.

We detected that 15 immune cells had remarkable causal effects on HCC. On the Monocyte panel, CX3CR1 on monocyte, CX3CR1 on CD14 + CD16 + monocyte and CX3CR1 on CD14 + CD16- monocyte could decrease the risk of HCC while CD40 on CD14 + CD16- monocyte could increase the risk of HCC. A research detected that patients with stable angina pectoris displayed significantly elevated CD14 + CD16 + CX3CR1 + monocyte counts compared with patients without vulnerable plaques [23]. Nine months after bare metal stent implantation after acute myocardial infarction, the number of CD14 + CD16 + CX3CR1 + monocytes in patients with restenosis was higher than that in patients without restenosis [24]. Moreover, Pang et al. found that IFNα-producing plasmacytoid dendritic cells drive CX3CR1 + myeloid-derived suppressor cells recruitment and result in neoplasm recurrence after surgical resection in HCC [25]. However, the role of these types of Monocyte cells in HCC has not been validated by biological experiments. On the Treg panel, CD28- CD8br %CD8br and CD127 on CD45RA + CD4 + could decrease the risk of HCC while CD39 on granulocyte and CD8 on CD39 + CD8br could increase the risk of HCC. Mei Sakuma et al. suggested that the change of CD45RA − CD27 + CD127 + central memory T cells frequency during nivolumab treatment might be a biomarker for predicting its therapeutic effect on esophageal squamous cell carcinoma patients [26]. A recent study indicated that high affinity new antigens trigger anti-tumor activity by activating tumor responsive CD39 + CD8 + T cells in hepatocellular carcinoma [27]. However, the role of CD28- CD8br %CD8br and CD39 on granulocyte in HCC has not been verified in HCC yet. On the TBNK panel, SSC-A on CD4 + , FSC-A on CD4 + and SSC-A on granulocyte could increase the risk of HCC. However, the role of SSC-A on CD4 + , FSC-A on CD4 + and SSC-A in the occurrence and development of HCC remains unclear. On the Maturation stages of T cell panel, Naive CD4 + %CD4 + and TD DN (CD4-CD8-) %T cell could decrease the risk of HCC while CD8 on EM CD8br could increase the risk of HCC.CD4 + T cells exert an integral role in immune responses and tissue homeostasis [28]. A recent study suggested that the number of CD4 + T cells displayed a remarkable impact on the survival and prognosis of HCC patients [29]. On the cDC panel, CD11c on granulocyte could increase the risk of HCC. Dendritic cells are the most powerful antigen-presenting cells, which could stimulate naive T cells and drive primary immune response [30]. Due to its capability to stimulate anti-tumor specific T cells, DCs vaccines presented a prospective adjuvant immunotherapy method for HCC patients [31].

The prognosis of HCC was extremely and complexly determined by a wide variety of factors. Precisely, factors like the inherent traits of the tumor itself, the therapeutic approaches, the patient-related elements, and additionally, the post-treatment follow-up and monitoring regimens, all had a crucial and close connection with the final prognosis. One research result indicated that among high-risk populations, compared with not conducting screening, screening for HCC was more effective in reducing the mortality rate of HCC and prolonging the overall survival period [32]. Besides, the portal venous coefficient and the hepatic arterial coefficient of the tumor before hepatectomy were highly valuable for independently forecasting the postoperative survival condition of patients suffering from HCC [33]. Lately, a good number of scholars have developed prognostic models through a series of technological means including gene analysis. Meanwhile, they have also taken part in the research on matters related to immunity, hoping to lend assistance to the prognostic evaluation and treatment of HCC [34, 35]. The findings of our research also implied that certain kinds of immune cells could serve as biomarkers for the risk of HCC, which could have brought about more efficient treatment options and more favorable prognoses.

Moreover, HCC was discovered to have causal effects on 21 immune cells. Notably, it is found that the existence of HCC were closely related to the increased levels of CD33 on Mo MDSC and reduced levels CD4 on EM CD4 + . A recent research indicated that high MDSC infiltration was an independent prognostic risk factor for overall survival and recurrence-free survival in HCC patients [36]. The expression of CD33 on activated macrophages was related to the disease progression and low survival rate of HCC patients [37]. Chaoul et al. found that the total CD4 + T cells and effector memory CD4 + T cells in HCC patients decreased compared with the healthy control groups [38]. Consistent with our findings, the presence of HCC was associated with decreased CD4 on EM CD4 + . However, the specific reasons remain to be studied. The diverse immune cells that exerted an influence on HCC and those that were influenced by HCC did not intersect in any way. This also served as a form of reverse validation, indicating the precise significance of the causal effect.

## Limitations

Nevertheless, our research has some weaknesses. First, we chose GWAS aggregated datasets with large immune characteristics and HCC sample sizes. However, the data of immune cells and HCC came from different researches, and there were certain differences in sample size and race, which may lead to some mistakes. The conclusions could not be generalized to other ethnic groups. Second, owing to the lack of individual-level data, we could not implement further hierarchical analysis of the population. Finally, instead of using FDR correction to assess the results, we utilized a loose threshold, which may add some false positives.

## Conclusions

The present study furnished valuable proof that immune cells could affect HCC risk via a comprehensive genetic method according to large-scale GWAS aggregated data. Our results expand the understanding of the immunology of HCC onset, providing a new theoretical basis for immunotherapy of HCC.

## Supplementary Information

Supplementary material 1

Supplementary material 2

## Data Availability

Data were provided within the manuscript.
